# Autosomal Dominant Polycystic Kidney Disease and Idiopathic Arginine Vasopressin Deficiency: A Peculiar Case Report of Accelerated Kidney Function Decline

**DOI:** 10.1177/20543581251378016

**Published:** 2025-09-28

**Authors:** Farah Wehbe, Mark Elliott, Myriam Farah

**Affiliations:** 1 Division of Nephrology, Department of Medicine, St Paul’s Hospital, Vancouver, BC, Canada; 2 Division of Nephrology, The University of British Columbia, Vancouver, Canada

**Keywords:** ADPKD, arginine vasopressin deficiency (AVP-D), antidiuretic hormone (ADH), desmopressin (DDAVP), ESKD

## Abstract

Autosomal dominant polycystic kidney disease (ADPKD) is a common genetic kidney disorder characterized by progressive cyst growth and kidney impairment. Arginine vasopressin deficiency (AVP-D) is a rare disorder resulting from reduced arginine vasopressin production, causing polyuria and thirst. The coexistence of ADPKD and AVP-D is rarely documented in the literature. We report what may be the first documented case of a patient diagnosed with ADPKD and idiopathic AVP-D. Initially managed with intranasal desmopressin, the patient’s kidney function declined earlier than expected based on her ADPKD, progressing to kidney failure at a low total kidney volume (836 mL). This paradoxical outcome suggests that while AVP-D may have initially slowed cyst growth, her uncontrolled AVP-D likely contributed to kidney function decline, presumably due to recurrent volume depletion and acute kidney injuries. This case highlights the need for individualized AVP-D management in ADPKD patients and reiterates AVP’s role in the complex pathophysiology of ADPKD progression.

## Abrégé

La polykystose rénale autosomique dominante (ADPKD) est une néphropathie génétique courante caractérisée par une croissance progressive de kystes et une insuffisance rénale. Le déficit en arginine vasopressine (d-AVP), ou diabète insipide central, est un trouble rare résultant d’une production réduite d’arginine vasopressine qui se manifeste par une polyurie et une soif excessive. La coexistence de ces deux pathologies est très peu décrite dans la littérature. Nous présentons ce qui semble être le premier cas rapporté d’une atteinte simultanée d’ADPKD et d’un d-AVP idiopathique. La patiente, sous traitement initial par desmopressine nasale, a présenté un déclin de la fonction rénale plus précocement par rapport à celui attendu dans l’évolution habituelle de l’ADPKD, et a progressé vers l’insuffisance rénale malgré un volume rénal total relativement faible (836 mL). Ce résultat paradoxal suggère que, si le d-AVP a pu freiner initialement la croissance des kystes, son caractère non contrôlé a probablement contribué au déclin de la fonction rénale, vraisemblablement en raison d’épisodes répétés de déplétion volémique et d’insuffisance rénale aiguë. Ce cas illustre le besoin d’une prise en charge individualisée du d-AVP chez les patients atteints d’ADPKD, en plus de confirmer le rôle de l’AVP dans la physiopathologie complexe de la progression de la maladie.

## Introduction

Autosomal dominant polycystic kidney disease (ADPKD) is a common genetic kidney disorder, affecting approximately 1 in 1000 people. It is mainly caused by variants in *PKD1* and *PKD2*, leading to numerous fluid-filled cysts in the kidneys. Elevated levels of the antidiuretic hormone, arginine vasopressin (AVP), in ADPKD patients have been associated with increased cyst growth and disease progression. AVP binds to vasopressin 2 receptors (V2R) on kidney tubular epithelial cells, triggering a signaling cascade that leads to cyclic adenosine monophosphate (cAMP) production, which promotes cyst fluid secretion and stimulates cell proliferation. ADPKD progression commonly includes progressive cyst growth, elevated total kidney volume (TKV), kidney function decline, hypertension, and urine concentration defects.^1^

AVP deficiency (AVP-D) is a rare endocrine disorder due to deficient AVP secretion, causing hypotonic polyuria and thirst. AVP-D can be idiopathic, hereditary, or secondary to hypothalamic or pituitary lesions.^2^

Urinary concentrating ability is decreased in ADPKD patients. Therefore, the coexistence of ADPKD and AVP-D poses significant diagnostic and therapeutic challenges. We describe a case of concurrent ADPKD and idiopathic AVP-D in a 52-year-old female patient who progressed to kidney failure with lower TKV than typically anticipated. While AVP-D may have initially provided a protective effect against cyst growth, treatment with desmopressin reversed this effect, and her uncontrolled AVP-D likely contributed to the accelerated kidney function decline through recurrent episodes of acute kidney injury (AKI).

## Presenting Concerns

The patient is a 52-year-old woman who was first diagnosed with ADPKD at age 21. She has a strong paternal family history of ADPKD resulting in kidney failure by ages 50-60, and a presumed *PKD1* variant. No imaging from her diagnosis is available; however, a renal ultrasound at age 29 revealed bilaterally enlarged kidneys (right: 16 cm, left: 14.7 cm). She had an uneventful pregnancy at age 30, but 6 months postpartum, she developed an acute onset of severe thirst and polyuria exceeding 6 liters/day.

## Clinical Findings

Physical examination was unremarkable, with blood pressure of 110/70 mm Hg, no postural hypotension, and heart rate of 72 beats per minute. Her polyuria was further evaluated through a water deprivation test. After 6 hours of water restriction, urine output was 6 liters, with urine osmolality of 171 mOsm/kg. After administering 2 mcg of intravenous desmopressin acetate, urine osmolality increased to 399 mOsm/Kg. Further laboratory tests demonstrated a serum osmolality of 298 mOsm/Kg, serum sodium level of 142 mmol/L, potassium 4.4 mmol/L, chloride 106 mmol/L, bicarbonate 24 mmol/L, urea 4.3 mmol/L, creatinine 71 µmol/L with estimated glomerular filtration rate (eGFR) of 105 mL/min/1.73 m^2^, calcium 2.08 mmol/L, and urinary albumin to creatinine ratio of 1.8 mg/mmol.

## Timeline

**Figure 1.**
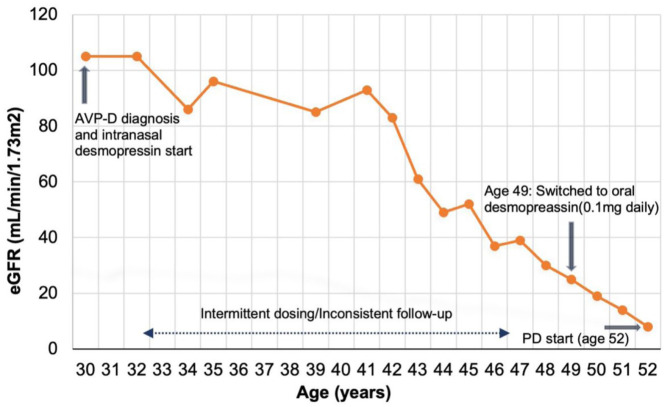
Trajectory of eGFR decline over time. *Note.* This graph illustrates the decline in the estimated glomerular filtration rate (eGFR mL/min//1.73m^2^) over time in our patient with ADPKD and AVP-D. eGFR was calculated using the CKD-EPI 2009 equation without race correction. The dashed line indicates the period of intermittent desmopressin dosing and inconsistent follow-up. PD = peritoneal dialysis.

A timeline of her imaging studies and interventions is provided in Table 1, and her eGFR over time is shown in Figure 1.

**Table 1.** Kidney size, Total Kidney Volume (TKV), and Estimated Glomerular Filtration Rate (eGFR) Over Time.

Age (years)	Kidney sizes (cm)	Imaging modality	Total Kidney volume (TKV), mL	Estimated glomerular filtration rate (eGFR) mL/min/1.73 m²	Comments
29	Right 16, Left 14.7	Ultrasound	-	105	-
30	Both 15	Ultrasound	-	105	AVP-D diagnosis and intranasal desmopressin initiation
35	Both 14.5	Ultrasound	-	96	
45	Right 14.2, Left 17	MRI	-	52	
49	Right 14.2, Left 13	MRI	610	25	Switched to oral desmopressin (0.1 mg daily)
51	Right 15.3, Left 12.1	MRI	836	14	ESKD reached
52	-	-	-	8	Onset of peritoneal dialysis

*Note.* Changes in kidney size (cm), total kidney volume (TKV, mL), and estimated glomerular filtration rate (eGFR, mL/min/1.73 m²) from age 29-52 years are shown. Key clinical events, including the diagnosis of Arginine vasopressin Deficiency (AVP-D) at age 30 and desmopressin initiation, are noted. eGFR was calculated using the CKD-EPI 2009 equation, based on serum creatinine levels.

## Diagnostic Focus and Assessment

Brain magnetic resonance imaging was normal without evidence of intracranial aneurysm, hemorrhage, or space-occupying lesions. Pituitary hormone levels, including thyroid-stimulating hormone, were normal, as were cortisol levels. Other acquired causes, such as Sheehan’s syndrome, autoimmune hypophysitis, and sarcoidosis, were excluded through endocrine assessment, pituitary imaging, chest X-ray, and laboratory tests. She was diagnosed with AVP-D, which was deemed to be idiopathic and unrelated to pregnancy.

She was started on 10 mcg of intranasal desmopressin, which led to more than 50% reduction in reported urine output and complete resolution of her thirst. However, due to the patient experiencing edema and bloating on this regimen, she adopted a self-dosing strategy with intermittent use of desmopressin whenever she experienced severe thirst, copious urine output, hypotension, or dizziness. She did not attend regular endocrinology follow-ups.

## Follow-up and Outcomes

The patient’s eGFR, calculated from creatinine using the CKD-EPI 2009 equation without race correction,^3^ remained between 83 and 105 mL/min/1.73 m² from ages 21 to 42. However, at age 45, her eGFR began to decline significantly (Figure 1). By age 49, when she became a patient at our ADPKD clinic, her eGFR had declined to 25 mL/min/1.73m². Initial magnetic resonance imaging revealed a TKV of 610 mL (height-adjusted TKV 370 mL/m), which represented little growth in kidney size compared to historic imaging dating back 20 years (Table 1).

The potential contribution of sub-optimal AVP-D management due to her desmopressin self-dosing strategy on kidney function decline was raised, given her discordant TKV and eGFR. She underwent a repeat endocrinology assessment, after which her desmopressin regimen was adjusted to daily oral dosing of 0.1 mg (Table 1). This change resulted in steady urine output and improved control of her blood pressure and thirst.

Her kidney function continued to decline from 25 to 14 mL/min/1.73 m^2^ (Figure 1) and repeat TKV 2 years later showed an increase to 836 mL (height-adjusted TKV 506 mL/m; Table 1). By age 52, she reached kidney failure and initiated peritoneal dialysis. Desmopressin was stopped due to the development of hyponatremia.

## Discussion

To our knowledge, this is the first reported case of idiopathic AVP-D in a patient with ADPKD. A literature search of PubMed, Google Scholar, and EMBASE identified only two cases of AVP-D in ADPKD patients: one case developed in a patient treated with maintenance dialysis 4 months after intracranial aneurysm rupture and clipping,^4^ and another case unmasked in a patient with a functional kidney transplant who had a history of ICA rupture.^5^ Our case is unique as the patient developed idiopathic AVP-D without prior intracranial intervention or trauma.

This case raises several important considerations. ADPKD is typically associated with elevated AVP, which is implicated in cyst growth and disease progression.^6^ Desmopressin, a synthetic analog of AVP, increases cyclic AMP levels in kidney epithelial cells through V2R signaling, a major driver of cyst growth by promoting cell proliferation and fluid secretion, which increases TKV.^7^ In contrast, AVP-D is characterized by reduced AVP levels, which would be expected to slow cyst growth and potentially delay disease progression.^2^

Our patient’s family history provides valuable context. Her father underwent a kidney transplant at age 62, her sister has a TKV of 1488 mL at age 54 with an eGFR of 30 mL/min/1.73m^2^, and her son has a TKV of 875 mL at age 23 with an eGFR of 120 mL/min/1.73 m^2^. In contrast, our patient at age 49 had a TKV of only 610 mL and an eGFR of 25 mL/min/1.73 m^2^, demonstrating a discordance between TKV growth and kidney function decline. We propose that years of vasopressin deficiency had stunted early TKV growth and that her eGFR decline was based on recurrent hypovolemic AKI events in the context of sporadic desmopressin exposure. Inconsistent follow-up likely led to under-recognized and undocumented AKI, limiting direct evidence of its role in kidney decline. With consistent desmopressin exposure over the next 2 years, her TKV grew from 610 mL to 836 mL, representing a rapid growth rate of 19% per year. She developed kidney failure at a similar age to other family members, but at a much lower TKV (836 mL).

Our case poses a more complicated hypothesis, highlighting the importance of factors in addition to progressive cyst growth on eGFR decline in patients with ADPKD. We propose that kidney function decline is linked to several additional pathophysiologic mechanisms. This is particularly evident in patients with *PKD2* variants, who can develop significant TKV enlargement with associated mass effect, but without corresponding kidney function loss.^8^

The Mayo imaging classification (MIC) system is a prognostic tool that relies on age and height-adjusted TKV to predict future risk of reaching end-stage kidney disease (ESKD) in ADPKD patients.^1^ Our patient’s MIC remained 1B at two serial time points, predicting a much better prognosis for her than for her son (currently classified 1E) and her sister (currently classified 1C).^9^ Patients in MIC 1B are reported to reach kidney failure at a median age of 65 years.^9^ Our patient reached kidney failure much earlier than predicted, with smaller kidneys than expected and compared to her family members. This demonstrates that prognostication in cases such as this is more complex than captured by the MIC system, given the unique influence of AVP-D.

Presently, our patient is treated with peritoneal dialysis and awaiting kidney transplantation. There are isolated reports of AVP-D symptoms unmasked in ESKD patients following kidney transplantation.^5,10^ We anticipate that she will develop symptoms of AVP-D post-transplantation and will require careful desmopressin administration to avoid deleterious impacts of polyuria and volume depletion on the allograft.

Our case report has a few limitations. One is the absence of historic cross-sectional imaging-based TKV before age 49, largely owing to evolving standards of practice and limited records from previous care settings. Nonetheless, we were able to obtain serial historical ultrasound-based kidney dimensions, providing important data on kidney size in the first five decades of life. Second, although her AVP-D was diagnosed based on clinical features, water deprivation test, and expert endocrinology assessment, AVP and copeptin levels were not available for further diagnostic clarification. Lastly, we do not have genetic testing results in this family, reflecting real-life clinical practice in the management of patients with ADPKD and a strong family history.

This case report underlines the extremely rare co-occurrence of idiopathic AVP-D and ADPKD. By presenting this case, we hope to raise awareness among physicians about the possibility of this co-occurrence and its potential impact on disease progression. It emphasizes the need for individualized AVP-D management in patients with ADPKD and reiterates the role AVP plays in the complex pathophysiology of ADPKD progression. It also exemplifies that standard prognostication tools may not be reliable in specific patients.
